# Effects of Reusing Baseline Volumes of Interest by Applying (Non-)Rigid Image Registration on Positron Emission Tomography Response Assessments

**DOI:** 10.1371/journal.pone.0087167

**Published:** 2014-01-28

**Authors:** Floris H. P. van Velden, Ida A. Nissen, Wendy Hayes, Linda M. Velasquez, Otto S. Hoekstra, Ronald Boellaard

**Affiliations:** 1 Department of Radiology & Nuclear Medicine, VU University Medical Center, Amsterdam, Noord-Holland, The Netherlands; 2 Exploratory Clinical and Translational Research, Bristol-Myers Squibb, Princeton, New Jersey, United States of America; NIH, United States of America

## Abstract

**Objectives:**

Reusing baseline volumes of interest (VOI) by applying non-rigid and to some extent (local) rigid image registration showed good test-retest variability similar to delineating VOI on both scans individually. The aim of the present study was to compare response assessments and classifications based on various types of image registration with those based on (semi)-automatic tumour delineation.

**Methods:**

Baseline (n = 13), early (n = 12) and late (n = 9) response (after one and three cycles of treatment, respectively) whole body [^18^F]fluoro-2-deoxy-D-glucose positron emission tomography/computed tomography (PET/CT) scans were acquired in subjects with advanced gastrointestinal malignancies. Lesions were identified for early and late response scans. VOI were drawn independently on all scans using an adaptive 50% threshold method (A50). In addition, various types of (non-)rigid image registration were applied to PET and/or CT images, after which baseline VOI were projected onto response scans. Response was classified using PET Response Criteria in Solid Tumors for maximum standardized uptake value (SUV_max_), average SUV (SUV_mean_), peak SUV (SUV_peak_), metabolically active tumour volume (MATV), total lesion glycolysis (TLG) and the area under a cumulative SUV-volume histogram curve (AUC).

**Results:**

Non-rigid PET-based registration and non-rigid CT-based registration followed by non-rigid PET-based registration (CTPET) did not show differences in response classifications compared to A50 for SUV_max_ and SUV_peak,_, however, differences were observed for MATV, SUV_mean_, TLG and AUC. For the latter, these registrations demonstrated a poorer performance for small lung lesions (<2.8 ml), whereas A50 showed a poorer performance when another area with high uptake was close to the target lesion. All methods were affected by lesions with very heterogeneous tracer uptake.

**Conclusions:**

Non-rigid PET- and CTPET-based image registrations may be used to classify response based on SUV_max_ and SUV_peak_. For other quantitative measures future studies should assess which method is valid for response evaluations by correlating with survival data.

## Introduction

Positron emission tomography/computed tomography (PET/CT) has been shown to be a valuable tool in oncology for monitoring response to treatment [1]. Volumes of interest (VOI) can be defined on the pre-treatment PET/CT scan and on consecutive (response) scans during or after treatment to measure changes (response) in metabolically active tumour volume (MATV). tracer uptake or uptake heterogeneity [2]. A 3 dimensional isocontour method at 50% of the maximum pixel value that corrects for local background (A50) is a highly reproducible method to define VOI (semi-)automatically [3]–[6]. Ideally baseline VOI should be projected onto the consecutive (response) scans to enable more efficient therapy efficacy assessment [7]. One practical issue with longitudinal PET/CT studies is that patient positioning between consecutive scans may vary, thereby inhibiting the direct reuse of baseline VOI for response scans. Image registration between consecutive scans is required to facilitate reuse of baseline VOI. These image registrations can be performed either rigidly or non-rigidly. Rigid image registration only allows for rotational and translational movements of the entire image, whereas non-rigid image registration allows for any type of local (elastic) deformations. A previous test-retest study showed that reusing baseline VOI by applying non-rigid and to lesser extent (local) rigid image registration has good repeatability, similar to delineating VOI on either scan separately [8]. However, in a test-retest setting, no changes in tumour shape, volume, tracer uptake and/or tracer uptake heterogeneity are expected, because these studies are acquired within a limited time frame and without administration of therapy. In a response monitoring setting, the interval between consecutive scans can be several weeks. For this reason, not only difference in patient positioning between consecutive PET/CT scans may pose a challenge for image registration strategies in longitudinal PET/CT studies, but also changes in tumour shape, volume, tracer uptake and tracer uptake heterogeneity, resulting from either treatment effects or progression of the disease [8], [9].

The purpose of the present study was to investigate the effects of reusing baseline VOI by (non-)rigid image registration strategies proposed previously [8] on PET/CT response assessments and response classifications by comparing the results to those obtained using A50 to delineate VOI on baseline and response scans separately.

## Materials and Methods

### Patient data

Baseline whole-body [^18^F]fluoro-2-deoxy-D-glucose ([^18^F]FDG) PET/CT studies were acquired for 13 patients (9 male, 4 female; age: 60±12 y; weight: 84±17 kg; height: 172±9 cm) with advanced colorectal carcinoma at five different sites [10]. Patients were only included if their double baseline studies demonstrated good repeatability [10]. All patients had received no therapy (chemotherapy, radiotherapy, or surgical treatment) for 2 weeks prior to the baseline scan. The patients were treated by BMS-582664 (brivanib alaninate) in combination with full-dose cetuximab (Erbitux), a monoclonal antibody targeting epidermal growth factor receptor. BMS-582664 is a selective dual inhibitor of fibroblast growth factor and vascular endothelial growth factor signalling, and is taken orally on a daily schedule [11]. Twelve patients underwent an early [^18^F]FDG PET/CT response scan after 1 cycle (day 15) of treatment, and nine patients a late [^18^F]FDG PET/CT response scan after 3 cycles (day 56). Patients fasted for at least 4 h prior to scanning and refrained from strenuous physical activity. Blood glucose levels were obtained for each patient prior to scanning and were within the normal range (5.6±1.0 mmol·l^−1^).

A static whole-body emission scan was started 84±32 min after injection of [^18^F]FDG (469±85 MBq). Prior to the emission scan, a (low dose) CT scan (120/130 kVp and 78–126 mAs) was acquired for attenuation correction purposes. All data were reconstructed according to local guidelines, which comply with published guidelines for quantitative [^18^F]FDG PET/CT studies [12]. Two patients were scanned on a Gemini PET/CT scanner (Philips Healthcare, Cleveland, OH, USA). PET images were reconstructed onto a 144×144 image matrix (voxel size: 4.0×4.0×4.0 mm) using a row action maximum likelihood algorithm with 2 iterations and 33 subsets. The corresponding CT images were reconstructed onto a 512×512 image matrix with a voxel size of 0.78×0.78×5.0 mm. Eleven patients were scanned on a Biograph PET/CT scanner (CTI/Siemens, Knoxville, TN, USA). PET images were reconstructed onto either 128×128 (voxel size: 5.2×5.2×2.4 mm, n = 4; or 5.3×5.3×3.4 mm, n = 6) or 168×168 (voxel size: 4.1×4.1×2.0 mm, n = 1) image matrices using an ordered-subsets expectation maximization algorithm with 2 to 4 iterations and 8 subsets. The corresponding CT images were reconstructed onto a 512×512 image matrix with a voxel size of 0.98×0.98×2.4 (n = 4), 0.98×0.98×2.5 (n = 6) or 0.98×0.98×4.0 (n = 1) mm. Following reconstruction, PET image data were expressed in standardized uptake values (SUV) by normalising voxel radioactivity concentrations to the injected dose and lean body mass [13]. All data were acquired as part of an ongoing clinical study [10], [11] approved by authorised medical ethical review committees (Georgetown University Oncology Institutional Review Board, University of South Florida Institutional Review Board, Western Institutional Review Board, University of Southern California School of Medicine Institutional Review Board and University Health Network Research Ethics Board), and written informed consent was obtained from each patient prior to inclusion in the study.

### Image registration strategies

All registrations were performed using Elastix (UMC Utrecht, The Netherlands) [14]. Various rigid and non-rigid strategies were evaluated based on various input data [8]:

- PET to PET image registration. This registration type takes functional information into account;
- CT to CT image registration. This registration takes anatomical information into account. The low dose CT scans were downsampled to the PET resolution prior to image registration to increase computational performance and to avoid issues with computer memory;
- CT to CT image registration, after which the transformation was used to initialize PET to PET registration (referred to as CTPET). This registration initially takes the anatomical followed by the functional information. This method was only used for (non-linear) non-rigid transformations, as (linear) rigid CTPET-based image registration would produce identical results to rigid PET-based image registration.

These various types of rigid and non-rigid image registration were applied on whole-body images, referred to as ‘global’. In addition, these various types of rigid image registration were also applied on selected whole-body images, cropped in such a way that they included slices with either the abdomen or lung. This method is referred to as ‘local’. In total, 7 different image registration strategies were investigated for response monitoring purposes. More details on the applied registration strategies and the corresponding settings for Elastix can be found in the literature [8].

### Data analysis

In total, 29 lesions were identified on the baseline scan located in the liver (n = 17), lung (n = 10), bone (n = 1) or pancreas (n = 1). For early response assessments, 27 lesions could be identified located in the liver (n = 15), lung (n = 10), pancreas (n = 1) or bone (n = 1). For late response assessments, 18 lesions could be identified located in the liver (n = 9), lung (n = 8) or pancreas (n = 1). VOI were drawn on baseline and both response scans using A50, resulting in baseline and (early and late) response VOI_A50_. In addition, baseline scans were registered onto the (early and late) response scans using the various registration strategies, after which baseline VOI_A50_ were transformed according to the transformation parameters obtained, resulting in VOI_registered_. For each VOI, maximum SUV (SUV_max_), peak SUV based on 1.2 cm diameter spherical VOI (SUV_peak_) [15], average SUV (SUV_mean_), MATV, total lesion glycolysis (TLG, calculated as product of SUV_mean_ and MATV) and area under a cumulative SUV-volume histogram (AUC) [2], [16] were obtained. AUC is a quantitative index of uptake heterogeneity, with lower AUC corresponding to a higher degree of (global) uptake heterogeneity [2], [17], [18]. SUV_mean_, MATV, TLG and AUC were not determined for VOI obtained using rigid image registration, due to its inability to change the shape or volume of a VOI. For all the quantitative measures, (early and late) responses were calculated as the values of the (early or late) response scans (obtained from either VOI_registered_ or response VOI_A50_) divided by the values of the baseline scan (obtained using baseline VOI_A50_) times 100%.

To assess the agreement between VOI_registered_ and response VOI_A50_, Dice similarity coefficients (DSC) were calculated between VOI_registered_ and response VOI_A50_ using 
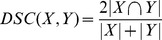
, where X denotes the volume of VOI_registered_, Y the volume of response VOI_A50_, and 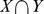 the overlap between the two volumes. A value of 0 indicates no overlap, whereas a value of 1 indicates perfect agreement. The level of agreement between responses obtained using A50 and each registration strategy was determined for each quantitative measure using intraclass correlation coefficients (ICC) with a two-way random single measures model (SPSS, Chicago, IL, USA). An ICC of 1 indicates a perfect agreement. Statistical significance was determined using a two-tailed paired Student's t-test, where p-values less than 0.05 were considered significant. Correlations between DSC and various values derived from MATV and AUC (absolute values of baseline and consecutive scans, and absolute responses) were assessed using squared Pearson's correlation coefficients (R^2^).

### Response classification

The obtained (early and late) responses for SUV_peak_ were classified using PET Response Criteria in Solid Tumors version 1.0 (PERCIST) [15] as progressive metabolic disease (PMD), stable metabolic disease (SMD), partial metabolic response (PMR) and complete metabolic response (CMR). PERCIST specifies that PMR requires greater than a 30% and a 0.8 g/ml decline in SUV_peak_ between the most intense lesions before as well as after treatment (not necessarily the same lesion); PMD requires > 30% and 0.8 g/ml increase in SUV_peak_ or new lesions; CMR is assigned when all metabolically active tumours have visually disappeared. Unlike PERCIST, classification was not performed per subject for the metabolically most active lesion only, but for each lesion individually. As CMR can be observed visually, CMR lesions were excluded. The response thresholds of SUV_peak_ were also used for SUV_max_. PERCIST does not specify response thresholds for SUV_mean_, TLG (only for PMD), MATV and AUC. Therefore, these thresholds were derived from retrospective test-retest data obtained using A50 [3]. These thresholds could then be used to classify responses in SUV_mean_, TLG, MATV as PMD, SMD and PMR, and observed responses in AUC as an increase in tracer uptake heterogeneity (IUH), stable tracer uptake heterogeneity (SUH) or a decrease in tracer uptake heterogeneity (DUH). The percentage response thresholds were obtained by calculating the mean test-retest value plus two times the standard deviation, rounded up to the next multiple of ten. The absolute response thresholds were obtained by calculating the mean absolute difference between test and retest values plus two times the standard deviation, rounded up to the tenth decimal place. More details on the used dataset can be found in [3]. Response thresholds derived from retrospective test-retest data were 30% with a minimum change of 1.6 g/ml, 110% with a minimum change of 11 ml, 10% with a minimum change of 0.06, and 110% with a minimum change of 28 g for SUV_mean_, MATV, AUC and TLG, respectively (table 1).

**Table 1:** Response thresholds derived from retrospective test-retest data.

	SUV_mean_	MATV	TLG	AUC
Mean test-retest variability (%)	8.2±7.4	29.8±39.3	31.1±36.3	2.6±2.5
Mean absolute difference^a^	0.4±0.6	2.2±4.1	8.4±9.6	0.02±0.02
Response threshold (%)	30	110	110	10
Absolute response threshold^a^	1.6	11	28	0.06

a Units were g/ml, ml and g for SUV_mean_, MATV and TLG, respectively.

Abbreviations: SUV, standardized uptake value; MATV, metabolically active tumour volume; TLG, total lesion glycolysis; AUC, area under a cumulative SUV-volume histogram curve.

## Results

### Overlap between VOI obtained using A50 and each registration strategy

**Figure 1:**
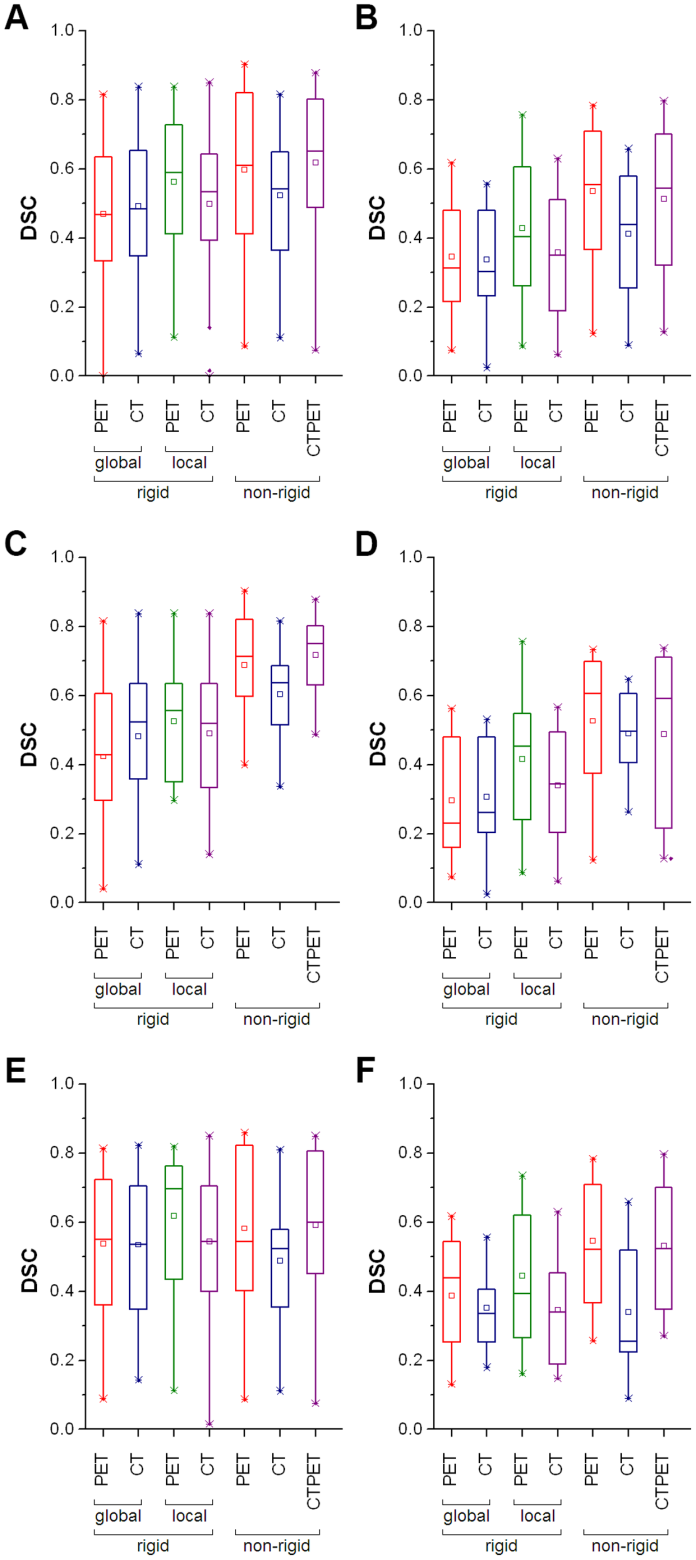
Similarity between volumes of interest (VOI) obtained using A50 and various registration strategies. Box plots of Dice similarity coefficients (DSC) for early (A, C, F) and late (B, D, G) response assessments. DSC were obtained using all VOI (A, B), lung VOI (C, D) or liver VOI (E, F). The mean is illustrated by a square, outliers by dots, and minimum and maximum values by crosses. Abbreviations: A50, 3 dimensional (semi-)automatic isocontour method at 50% of the maximum pixel value that corrects for local background.

**Figure 2:**
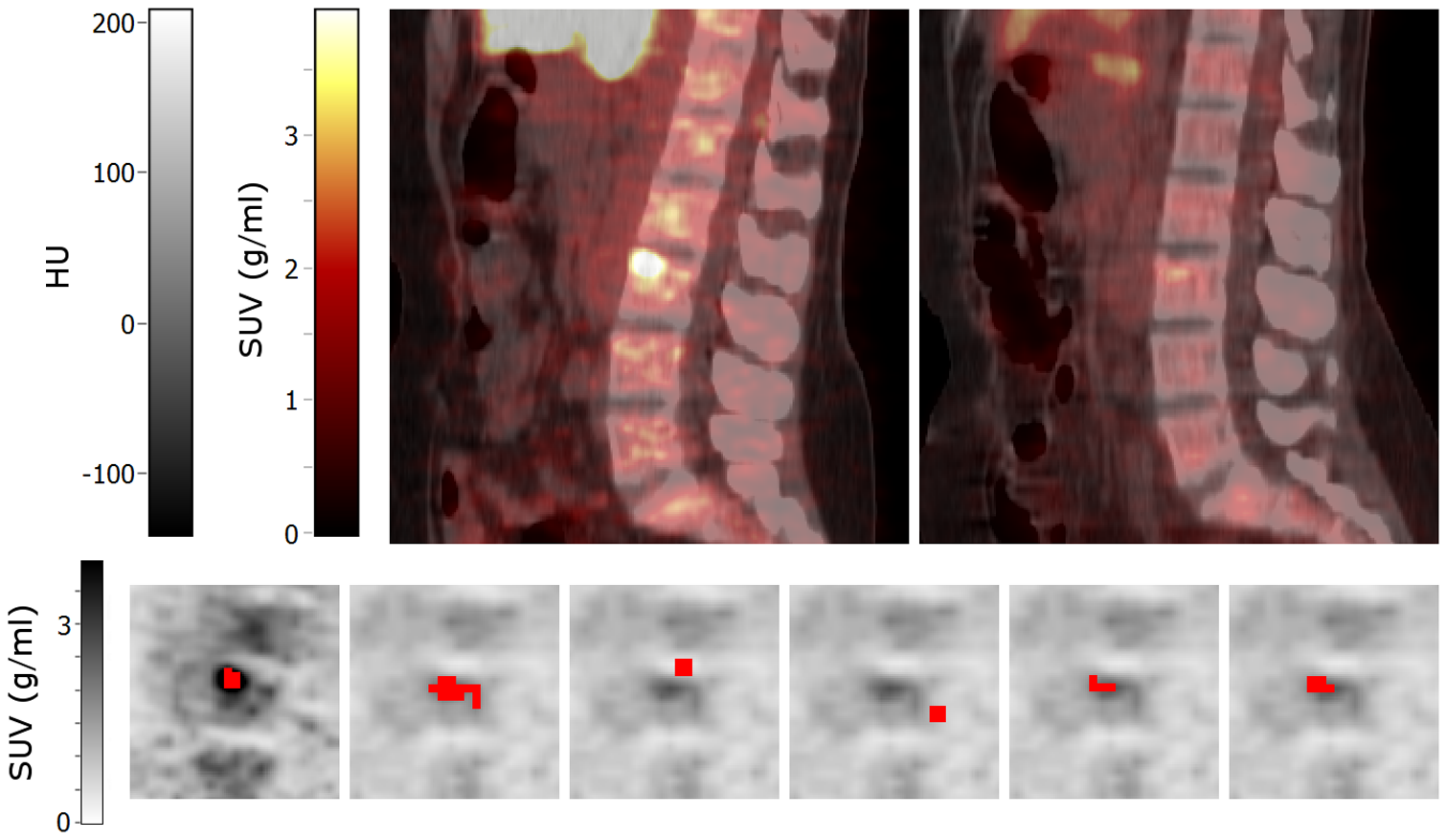
Sagittal images of a patient with a bone metastasis. Top row: baseline (left) and early response (right) PET/CT images. Bottom row: volumes of interest (shown in red) projected onto the baseline (first image) and early response scans (other images) that were obtained using (from left to right): A50 defined on baseline scan, A50 defined on early response scan, and global rigid PET, local rigid CT, non-rigid PET and non-rigid CTPET image registration. All images are shown using the same colour scales. Abbreviations: SUV, standardized uptake values; HU, hounsfield units; A50, 3 dimensional (semi-)automatic isocontour method at 50% of the maximum pixel value that corrects for local background.

Both non-rigid PET and CTPET registration showed the highest median DSC for early and late response assessments (early response assessments: 0.61 and 0.65, respectively; late response assessments: 0.55 and 0.54, respectively). For early response assessments, local rigid PET registration also showed a high median DSC (0.59). All registration strategies showed a decrease in median DSC from 9% (non-rigid PET registration) up to 38% (global rigid CT registration) between early and late response assessments (figures 1A and 1B, respectively). One VOI, located in bone, did not show overlap between A50 and global rigid PET or local rigid CT registration, and is illustrated in figure 2.

In general, DSC values obtained from lung VOI were significantly higher for non-rigid image registration compared to (local) rigid registration (p<0.04, figures 1C and 1D), except in early response assessments using local PET registration compared to non-rigid PET registration (p = 0.10). For liver VOI (figures 1E and 1F), non-rigid PET registration showed higher DSC values compared to (local) rigid PET registration in late response assessments (p<0.01), whereas non-rigid CT registration showed significantly lower DSC compared to local CT registration in early response assessments (p<0.05). Other results obtained for liver VOI were insignificant (p>0.12).

**Figure 3:**
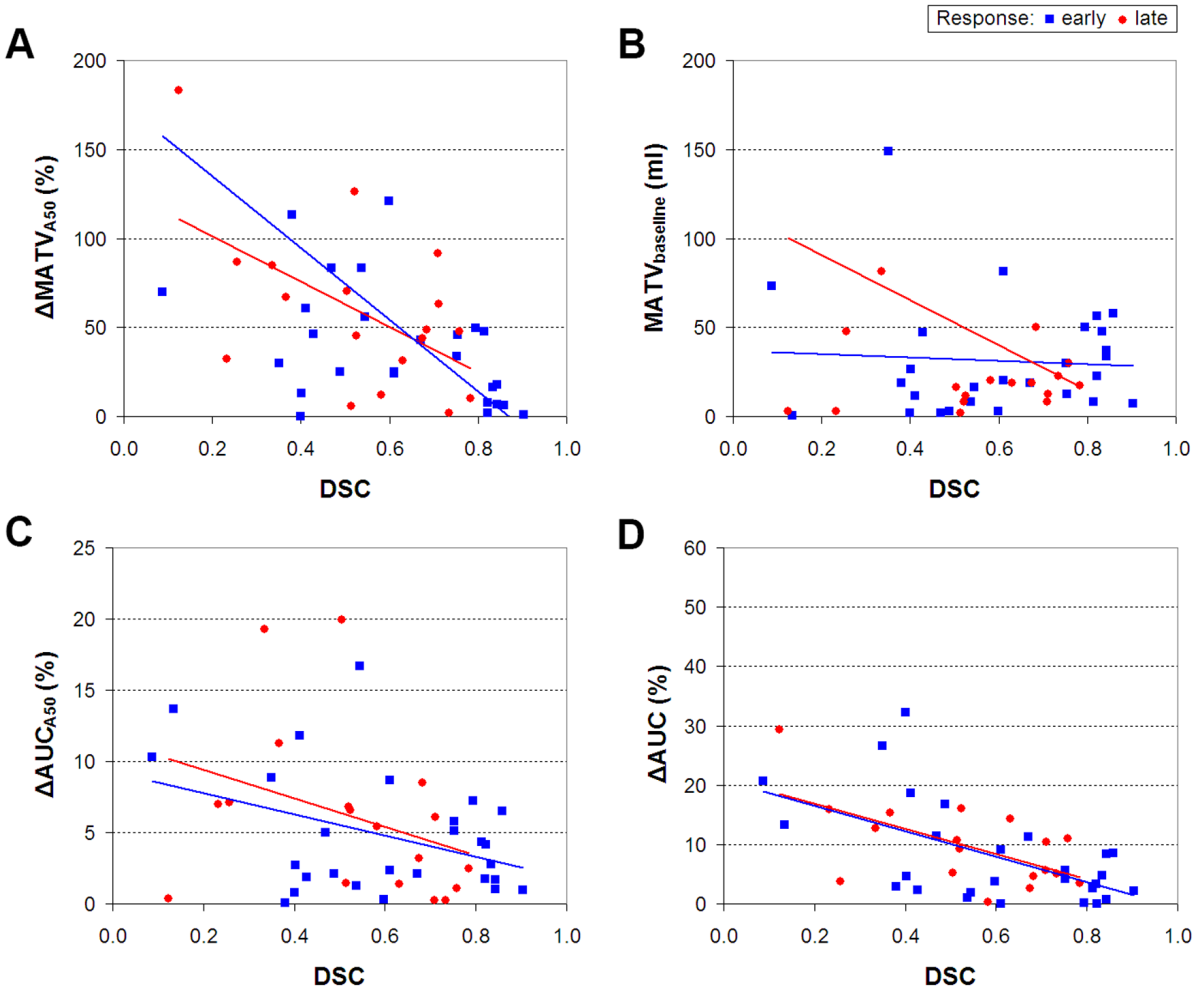
Correlation between Dice similarity coefficients and metabolically active tumour volume or tracer uptake heterogenity. Correlation between Dice similarity coefficients (DSC) obtained using non-rigid PET registration and (A) absolute MATV response values obtained using A50, (B) absolute baseline MATV values obtained using A50, (C) absolute AUC response values obtained using A50, (D) absolute AUC response values obtained using non-rigid PET registration. The two lines represent the trend lines. Note that one data point for late response assessments falls outside the scale of subfigure B (DSC: 0.37, MATV: 500 ml). Abbreviations: MATV, metabolically active tumour volume; AUC, area under a cumulative SUV-volume histogram curve; A50; 3 dimensional (semi-)automatic isocontour method at 50% of the maximum pixel value that corrects for local background.

For early response assessments, there was a weak relationship between DSC and the absolute MATV response values obtained from either A50 or the registration strategy itself (table 2; R^2^: 0.20–0.42), except for non-rigid CT registration that showed no relationship (R^2^:<0.16). In late response assessments, only non-rigid PET registration showed a weak relationship between DSC and absolute MATV response values (R^2^: 0.31), all other methods did not show a relationship (R^2^:<0.19). Only non-rigid PET and CTPET registration showed a moderate relationship between DSC and the absolute AUC response values obtained from the registration strategy itself for both response assessments (R^2^: 0.31–0.37). All other values investigated (table 2) generally showed, no relationship with DSC. Some typical scatter plots for non-rigid PET registration are shown in figure 3.

**Table 2:** Correlation (R^2^) of DSC with MATV, AUC or the absolute differences in MATV or AUC between baseline and response scans.

			Data obtained using A50			Data obtained using registration strategies	
			Absolute values of			Absolute values of	
Response assessment	Measure	Registration strategy	Baseline scan	Response scan	Absolute responses	Response scan	Absolute responses
Early	MATV	Global rigid PET	0.02	0.11	0.24	-	-
		Global rigid CT	0.02	0.10	0.26	-	-
		Local rigid PET	0.00	0.08	0.20	-	-
		Local rigid CT	0.01	0.08	0.29	-	-
		Non-rigid PET	0.00	0.01	0.29	0.02	0.24
		Non-rigid CT	0.01	0.00	0.16	0.01	0.03
		Non-rigid CTPET	0.02	0.00	0.28	0.00	0.42
	AUC	Global rigid PET	0.05	0.08	0.11	-	-
		Global rigid CT	0.16	0.07	0.06	-	-
		Local rigid PET	0.04	0.06	0.11	-	-
		Local rigid CT	0.07	0.04	0.11	-	-
		Non-rigid PET	0.09	0.05	0.15	0.10	0.33
		Non-rigid CT	0.02	0.06	0.18	0.11	0.31
		Non-rigid CTPET	0.00	0.00	0.18	0.08	0.33
Late	MATV	Global rigid PET	0.01	0.00	0.04	-	-
		Global rigid CT	0.01	0.00	0.04	-	-
		Local rigid PET	0.06	0.03	0.19	-	-
		Local rigid CT	0.05	0.03	0.05	-	-
		Non-rigid PET	0.05	0.00	0.31	0.03	0.02
		Non-rigid CT	0.10	0.06	0.18	0.09	0.08
		Non-rigid CTPET	0.03	0.00	0.19	0.02	0.01
	AUC	Global rigid PET	0.04	0.23	0.05	-	-
		Global rigid CT	0.10	0.12	0.00	-	-
		Local rigid PET	0.04	0.00	0.05	-	-
		Local rigid CT	0.00	0.11	0.10	-	-
		Non-rigid PET	0.02	0.19	0.11	0.10	0.37
		Non-rigid CT	0.00	0.02	0.12	0.08	0.11
		Non-rigid CTPET	0.09	0.34	0.08	0.09	0.31

Abbreviations: DSC, Dice similarity coefficient; MATV, metabolically active tumour volume; AUC, area under a cumulative SUV-volume histogram curve; A50, 3 dimensional (semi-)automatic isocontour method at 50% of the maximum pixel value that corrects for local background.

### Effects on response values

**Figure 4:**
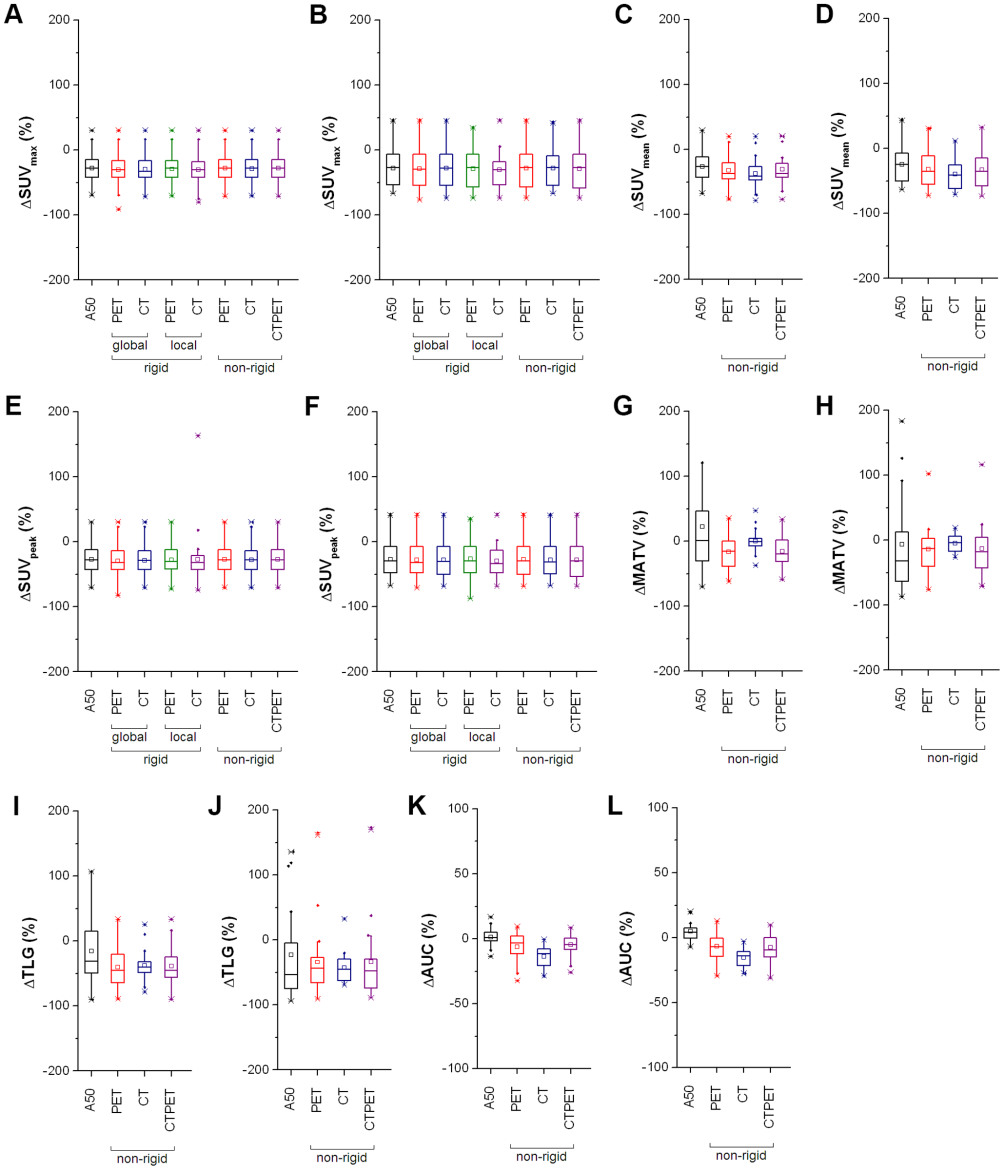
Effects on responses of various quantitative measures obtained using A50 and various registration strategies. Box plots illustrating the effects of various registration strategies on early (A, C, E, G, I and K) and late (B, D, F, H, J and L) responses derived from maximum standardized uptake value (SUV_max_; A, B), SUV_mean_ (C, D), SUV_peak_ (E, F), metabolically active tumour volume (MATV; G, H), total lesion glycolysis (TLG; I, J) or area under a cumulative SUV-volume histogram curve (AUC; K, L). Responses were calculated as the values of the (early or late) response scans divided by the values of the baseline scan times 100%. The mean is illustrated by a square, outliers by dots, and minimum and maximum values by crosses. Note that one data point for A50 falls outside the scale of subfigure G (447%). Abbreviations: A50, 3 dimensional (semi-)automatic isocontour method at 50% of the maximum pixel value that corrects for local background.

Absolute values of various quantitative PET measures obtained using A50 are listed in table 3. Median response values obtained using A50 and the various registration strategies are shown in figure 4. For both response assessments, SUV_max_ and SUV_peak_ response values derived from all registration strategies showed an almost perfect agreement with corresponding SUV_max_ and SUV_peak_ response values derived from A50 (table 4, ICC:>0.921), except for local rigid CT registration in early response assessments (ICC: 0.616). However, only non-rigid PET and CTPET image registration showed no significant differences in SUV_max_ and SUV_peak_ response values compared to those obtained from A50 (p>0.056). In addition, an almost perfect agreement was observed between SUV_mean_ response values derived from A50 and from non-rigid PET or CTPET registration (ICC:>0.923), but the observed differences were significant (p<0.011). Poor to moderate agreement was found between MATV, TLG and AUC response values derived from A50 and from non-rigid PET or CTPET registration (ICC: 0.034–0.763). One lesion (outlier in figure 4G) showed a large increase in MATV (447%) for A50 in the early response assessment and is illustrated in figure 2.

**Table 3:** Absolute values of various quantitative measures obtained with A50.

	Absolute values^a^					
	Baseline		Early response		Late response	
Measure	Median	Range	Median	Range	Median	Range
SUV_max_	13	3.6–21	8.4	2.7–27	5.6	1.7–22
SUV_peak_	11	2.6–19	7.0	2.0–25	4.5	1.4–18
SUV_mean_	9.7	2.8–15	6.0	2.2–19	4.3	1.4–15
MATV	20	0.6–500	23	1.7–104	13	1.8–166
TLG	163	3.8–2889	94	3.8–1018	55	2.6–774
AUC	0.82	0.71–0.98	0.84	0.72–0.97	0.85	0.78–0.98

a Units were g/ml, g/ml, g/ml, ml and g for SUV_max_, SUV_peak_, SUV_mean_, MATV and TLG, respectively.

Abbreviations: SUV, standardized uptake value; MATV, metabolically active tumour volume; TLG, total lesion glycolysis; AUC, area under a cumulative SUV-volume histogram curve; A50, 3 dimensional (semi-)automatic isocontour method at 50% of the maximum pixel value that corrects for local background.

**Table 4:** ICC and p-values calculated from response data obtained with various registration strategies and A50.

		Early response assessment				Late response assessment			
Measure	Registration strategy	P-value	ICC	Lower 95% CI	Upper 95% CI	P-value	ICC	Lower 95% CI	Upper 95% CI
SUV_max_	Global rigid PET	0.044^a^	0.965	0.920	0.984	0.067	0.993	0.979	0.997
	Global rigid CT	0.024^a^	0.986	0.964	0.994	0.104	0.997	0.991	0.999
	Local rigid PET	0.037^a^	0.993	0.984	0.997	0.053	0.993	0.979	0.997
	Local rigid CT	0.018^a^	0.975	0.936	0.989	0.281	0.938	0.846	0.976
	Non-rigid PET	0.060	0.999	0.997	0.999	0.180	0.998	0.995	0.999
	Non-rigid CT	0.053	0.995	0.989	0.998	0.106	0.997	0.993	0.999
	Non-rigid CTPET	0.128	0.999	0.997	0.999	0.116	0.997	0.990	0.999
SUV_peak_	Global rigid PET	0.048^a^	0.962	0.914	0.983	0.086	0.998	0.995	0.999
	Global rigid CT	0.031^a^	0.984	0.962	0.993	0.013^a^	0.999	0.994	1.000
	Local rigid PET	0.670	0.978	0.952	0.990	0.820	0.921	0.801	0.970
	Local rigid CT	0.989	0.616	0.310	0.806	0.297	0.923	0.810	0.970
	Non-rigid PET	0.056	1.000	0.999	1.000	0.170	1.000	0.999	1.000
	Non-rigid CT	0.051^a^	0.998	0.996	0.999	0.030^a^	0.999	0.997	1.000
	Non-rigid CTPET	0.208	1.000	1.000	1.000	0.105	0.999	0.996	0.999
SUV_mean_	Non-rigid PET	0.002^a^	0.923	0.762	0.970	<0.001^a^	0.945	0.524	0.986
	Non-rigid CT	<0.001^a^	0.853	0.131	0.957	<0.001^a^	0.818	−0.042	0.956
	Non-rigid CTPET	0.011^a^	0.939	0.841	0.974	<0.001^a^	0.932	0.488	0.982
MATV	Non-rigid PET	0.057	0.034	−0.295	0.380	0.616	0.456	−0.009	0.757
	Non-rigid CT	0.218	0.146	−0.229	0.488	0.920	−0.035	−0.524	0.443
	Non-rigid CTPET	0.046^a^	0.140	−0.197	0.469	0.658	0.393	−0.091	0.722
TLG	Non-rigid PET	0.012^a^	0.306	−0.034	0.597	0.319	0.763	0.479	0.903
	Non-rigid CT	0.015^a^	0.307	−0.034	0.598	0.176	0.407	−0.032	0.722
	Non-rigid CTPET	0.012^a^	0.373	0.025	0.648	0.351	0.747	0.449	0.896
AUC	Non-rigid PET	0.001^a^	0.160	−0.124	0.459	<0.001^a^	0.238	−0.113	0.597
	Non-rigid CT	<0.001^a^	0.026	−0.132	0.150	<0.001^a^	0.058	−0.055	0.276
	Non-rigid CTPET	0.002^a^	0.142	−0.143	0.445	<0.001^a^	0.194	−0.113	0.543

a Statistically significant difference (P<0.05).

Abbreviations: SUV, standardized uptake value; MATV, metabolically active tumour volume; TLG, total lesion glycosysis; AUC, area under a cumulative SUV-volume histogram curve; A50, 3 dimensional (semi-)automatic isocontour method at 50% of the maximum pixel value that corrects for local background; PET, positron emission tomography; CT, computed tomography; ICC, intraclass correlation coefficient; CI, confidence interval; VOI, volume of interest.

### Effects on response classifications

**Figure 5:**
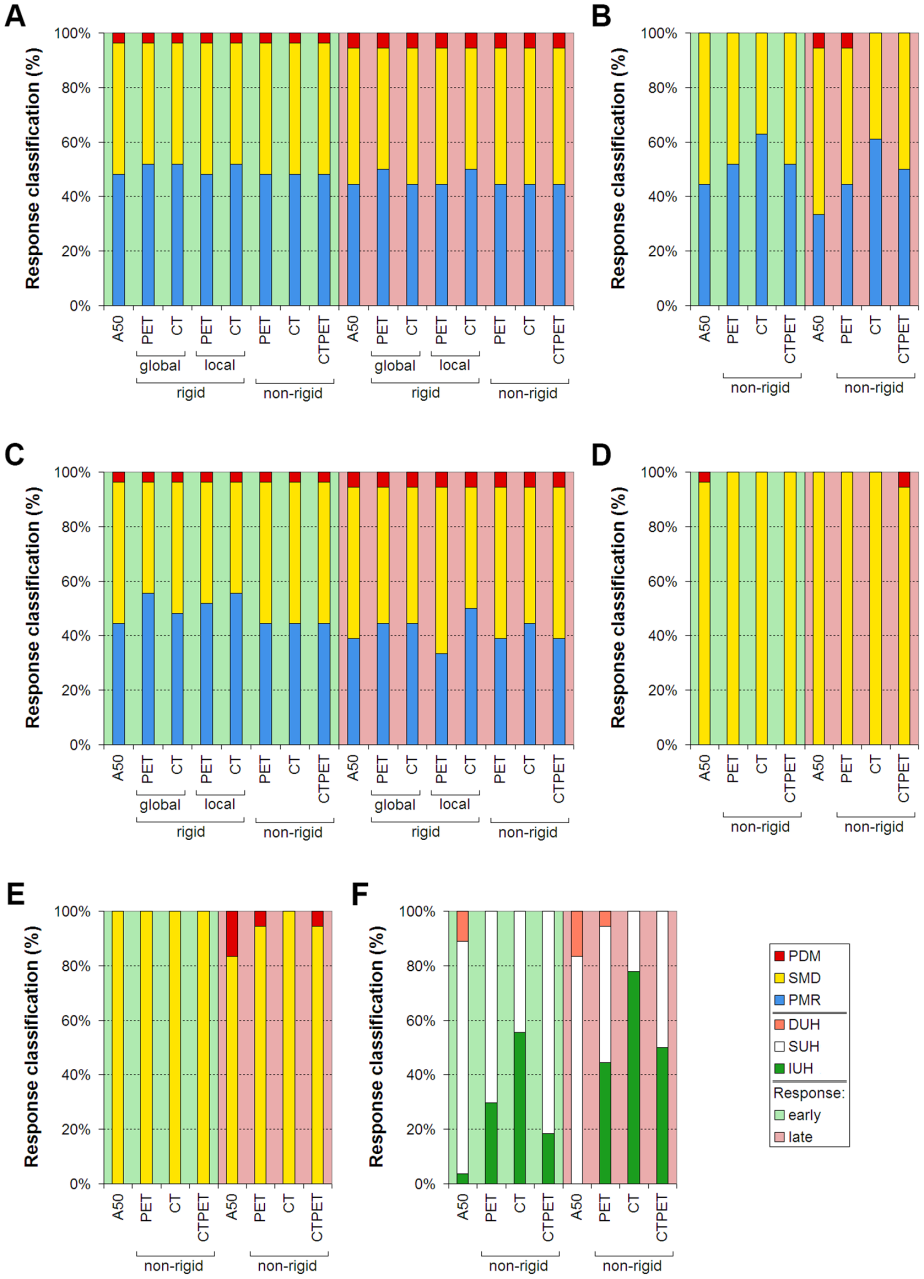
Response classifications for early and late response assessments. Response classifications for early (left part of each subfigure) and late (right part of each subfigure) response assessments based on maximum standardized uptake value (SUV_max_; A), SUV_mean_ (B), SUV_peak_ (C), metabolically active tumour volume (MATV; D), total lesion glycolysis (TLG; E) or area under a cumulative SUV-volume histogram curve (AUC; F). The response values were obtained using A50, local or global rigid image registration, or non-rigid image registration. Abbreviations: PMD, progressive metabolic disease; SMD, stable metabolic disease; PMR, partial metabolic response; IUH, an increase in tracer uptake heterogeneity; SUH, stable tracer uptake heterogeneity; DUH, a decrease in tracer uptake heterogeneity; A50, 3 dimensional (semi-)automatic isocontour method at 50% of the maximum pixel value that corrects for local background.

**Figure 6:**
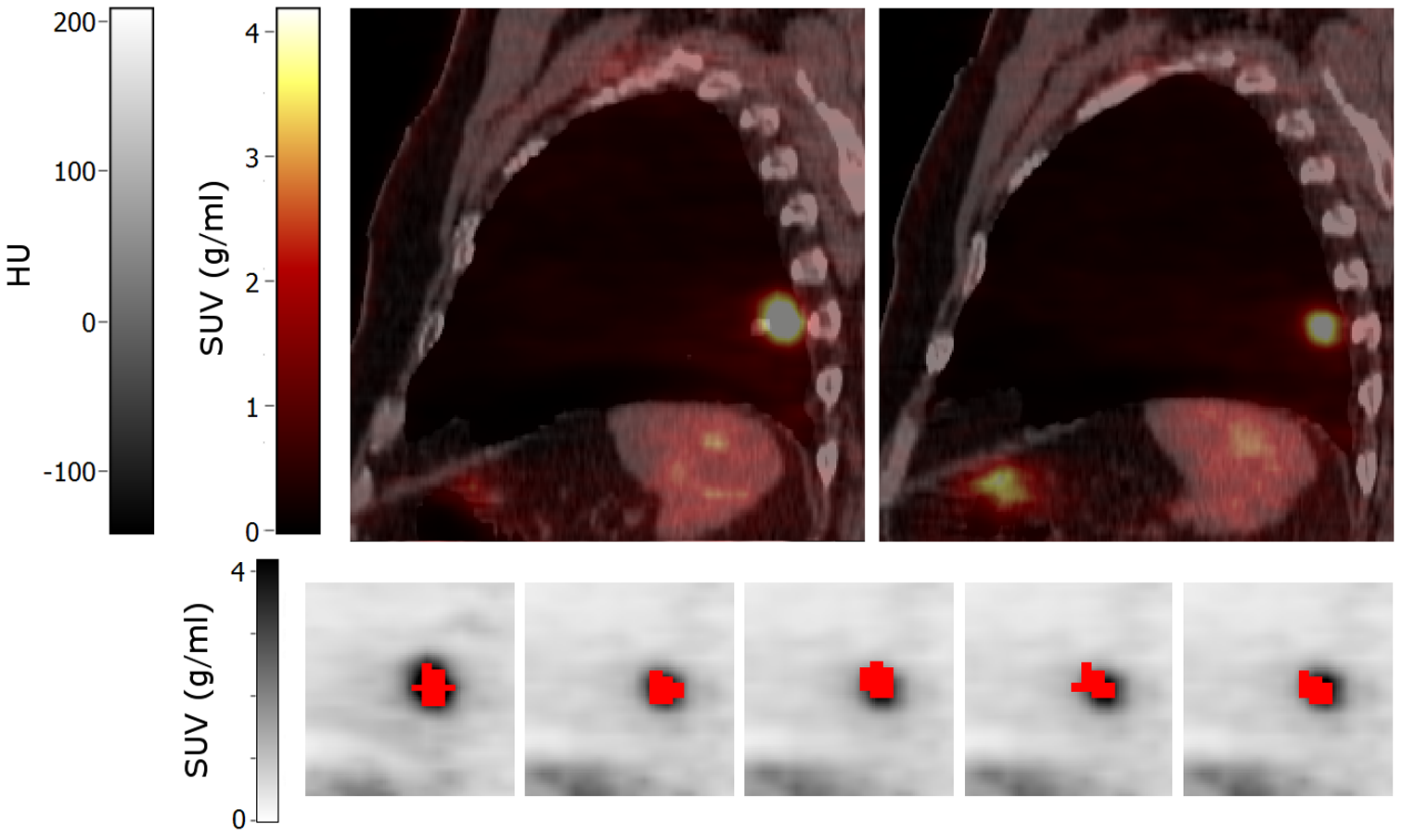
Sagittal images of a patient with a small lung metastasis. Top row: baseline (left) and early response (right) PET/CT images. Bottom row: volumes of interest (shown in red) projected onto the baseline (first image) and early response scans (other images) that were obtained using (from left to right): A50 defined on baseline scan, A50 defined on early response scan, and local rigid PET, non-rigid PET and non-rigid CTPET image registration. All images are shown using the same colour scales. Abbreviations: SUV, standardized uptake values; HU, hounsfield units; A50, 3 dimensional (semi-)automatic isocontour method at 50% of the maximum pixel value that corrects for local background.

**Figure 7:**
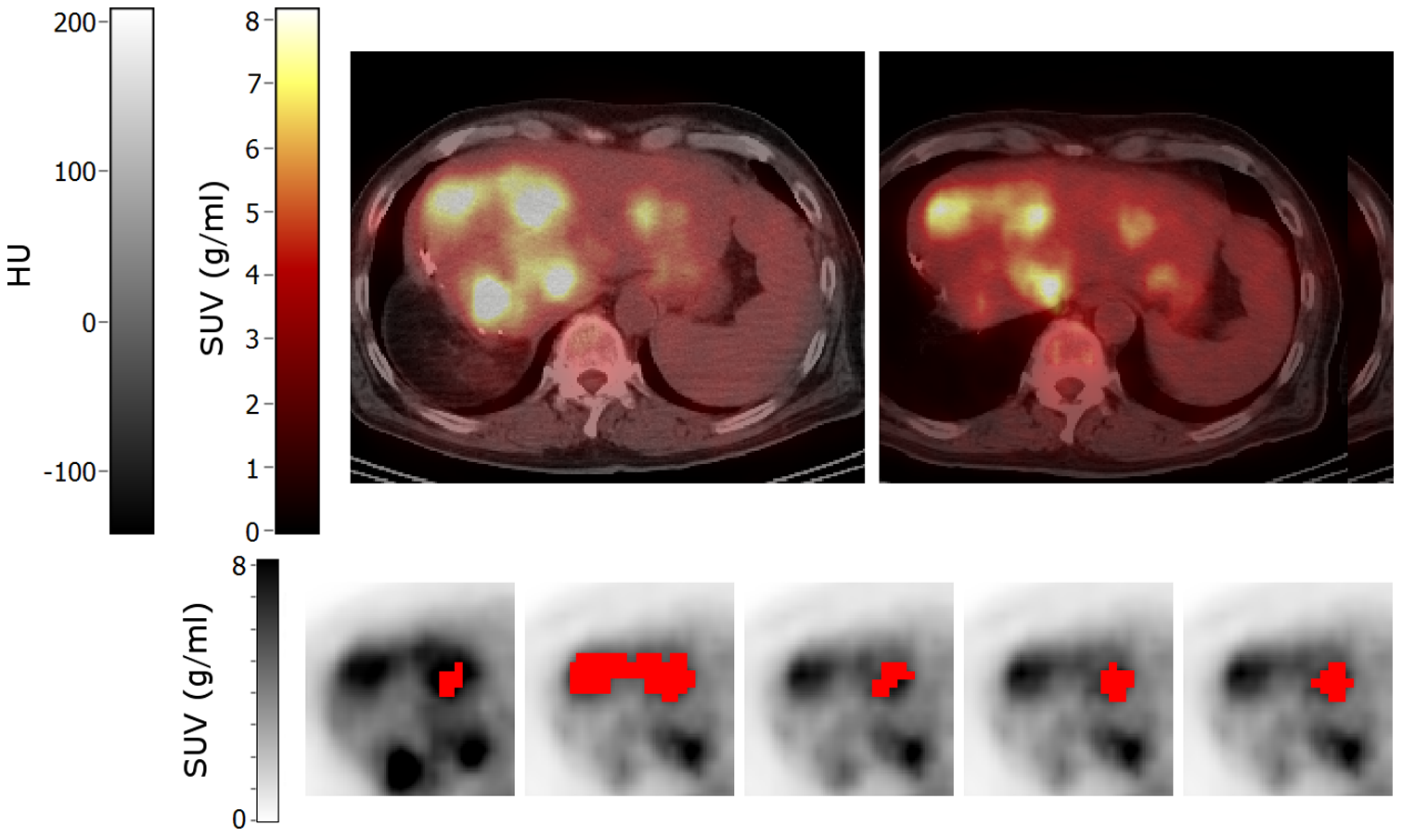
Axial images of a patient with liver metastases. Top row: baseline (left) and early response (right) PET/CT images. Bottom row: volumes of interest (shown in red) projected onto the baseline (first image) and early response scans (other images) that were obtained using (from left to right): A50 defined on baseline scan, A50 defined on early response scan, and local rigid PET, non-rigid PET and non-rigid CTPET image registration. All images are shown using the same colour scales. Abbreviations: SUV, standardized uptake values; HU, hounsfield units; A50, 3 dimensional (semi-)automatic isocontour method at 50% of the maximum pixel value that corrects for local background.

**Figure 8:**
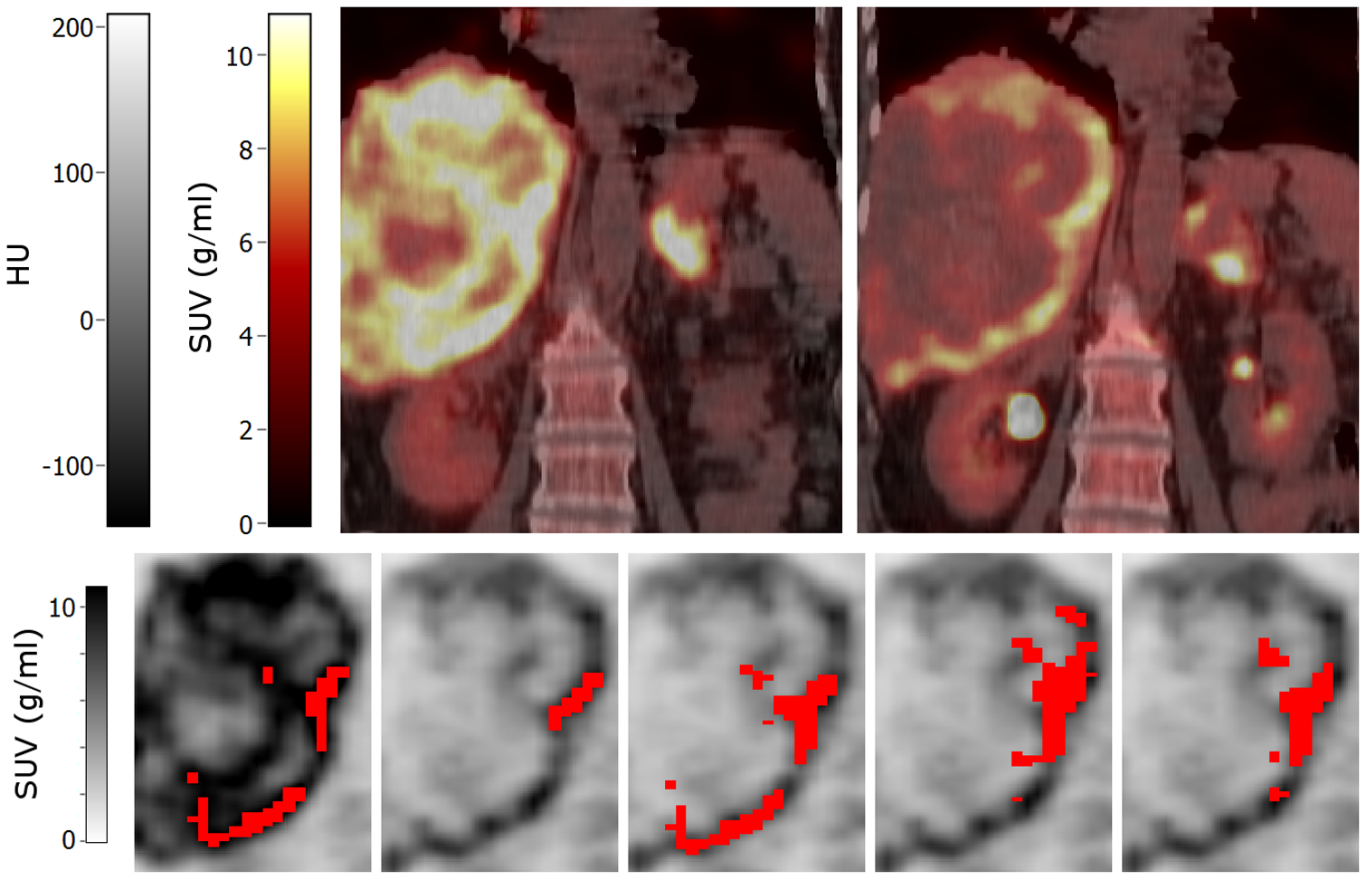
Coronal images of a patient with a large liver metastasis showing heterogeneous tracer uptake. Top row: baseline (left) and early response (right) PET/CT images. Bottom row: volumes of interest (shown in red) projected onto the baseline (first image) and early response scans (other images) that were obtained using (from left to right): A50 defined on baseline scan, A50 defined on early response scan, and local rigid PET, non-rigid PET and non-rigid CTPET image registration. All images are shown using the same colour scales. Abbreviations: SUV, standardized uptake values; HU, hounsfield units; A50, 3 dimensional (semi-)automatic isocontour method at 50% of the maximum pixel value that corrects for local background.

**Figure 9:**
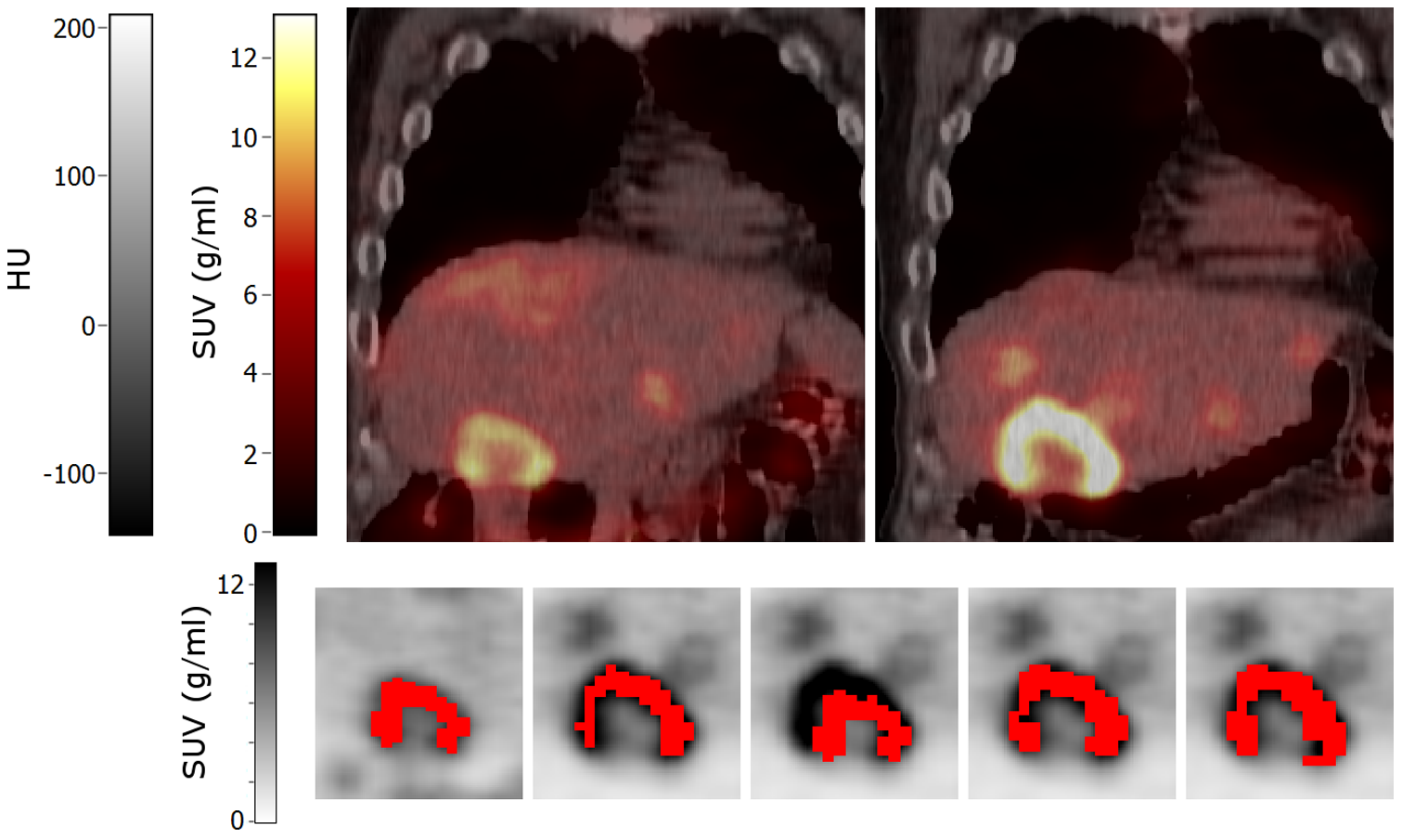
Coronal images of a patient with a liver metastasis that showed an increased metabolically active tumour volume. Top row: baseline (left) and late response (right) PET/CT images. Bottom row: volumes of interest (shown in red) projected onto the baseline (first image) and late response scans (other images) that were obtained using (from left to right): A50 defined on baseline scan, A50 defined on late response scan, and local rigid PET, non-rigid PET and non-rigid CTPET image registration. All images are shown using the same colour scales. Abbreviations: SUV, standardized uptake values; HU, hounsfield units; A50, 3 dimensional (semi-)automatic isocontour method at 50% of the maximum pixel value that corrects for local background.

Only non-rigid PET and CTPET registrations showed no differences in response classifications compared to A50 for SUV_max_ and SUV_peak_ (figure 5). However, for MATV, SUV_mean_ and TLG, compared with A50, non-rigid PET and CTPET registration showed in general more PMR and less SMD (up to 17%) or more SMD and less PMD (up to 11%). Moreover, non-rigid PET and CTPET registration showed more IUH and less SUH and/or DUH for AUC compared with A50 (up to 50%). Non-rigid CTPET and PET registration seemed to miscategorise response using SUV_mean_ and AUC for small lung lesions (<2.8 ml, figure 6), whereas A50 seemed to miscategorise response using MATV when another lesion with high uptake was close to the target lesion (figure 7). All methods seem to be affected by lesions with visually (increased) heterogeneous tracer uptake (figure 8). Three lesions showed deviating classifications between A50 and non-rigid CTPET and/or PET registration for two or more quantitative measures (TLG, SUV_mean_, MATV and/or AUC) in late response assessments that was caused by a slightly larger or smaller volume for the VOI obtained with CTPET and PET compared to the VOI obtained with A50 (figure 9). Their SUV_max_ changed by −4%, 46% and −23% to 15, 22 and 7 g/ml. For AUC, an additional 13 lesions showed deviating classifications between A50 and non-rigid CTPET and/or PET registration when lesions were very small (<5.0 ml, six lesions) or had a slightly larger or smaller volume for the VOI obtained with CTPET and PET compared to the VOI obtained with A50 (seven lesions).

## Discussion

This is the first study to investigate the effects of reusing baseline VOI by (non-)rigid image registration strategies on PET/CT response classifications and to compare these results to those obtained using VOI delineated on baseline and response scans separately. Out of all rigid registration strategies, local rigid PET registration showed the most similar performance to A50 for both response assessments. Nevertheless, local rigid PET registration showed one deviating response classification from A50 for SUV_peak_ in early response assessments (located in liver, figure 7) and two in late response assessments (located in liver and lung). Thus, (local) rigid image registration should not be applied to reuse baseline VOI for response classifications. These results are consistent with published data [9] whereby rigid registration provided imperfect alignment of breast tissue between longitudinal breast cancer PET/CT studies.

Non-rigid CT registration showed a poorer performance compared to non-rigid PET registration in both response assessments and to local CT registration for liver VOI in early response assessments. As discussed in a previous study [8], CT image registration may be improved by using respiratory gating [19] or intermodality image registration to correct for small residual misalignments between CT and PET (figure 6) [20], [21], or by using the original CT images that were not downsampled to the PET resolution. Although not shown for non-rigid CT registrations, reducing pixel resolution has little effect on the performance of rigid CT registration and can be used to speed up the algorithm without loss of accuracy [22].

These results indicate that non-rigid PET and CTPET registration may be used to classify response based on SUV_max_ and SUV_peak_. These results were consistent with results reported by De Moor et al. [7] showing that non-rigid image registration could be used to access therapy using PET more efficiently. However, differences in response classification were observed for MATV, SUV_mean_, TLG and AUC. For MATV, SUV_mean_ and TLG, differences were noted for small lung lesions (figure 6) or when another high uptake area or lesion was close to the target lesion (figures 2 and 7). In addition, all methods seem to be affected when a lesion showed (increased) heterogeneous tracer uptake (figure 8). Furthermore, some lesions showed a larger or smaller VOI in A50 compared to non-rigid PET or CTPET registration in late response assessment that had no apparent cause (figure 9). For AUC, an additional 13 lesions showed deviating classification non-rigid CTPET and/or PET registration and A50 when lesions were very small (<5.0 ml) or had a slightly larger or smaller volume for the VOI obtained with CTPET and PET compared to the VOI obtained with A50.

The registration of small lung lesions may be hampered by the limited registration parameters used in this study. As previously reported [8], registration parameters of the registration software (Elastix) could be adjusted to allow higher DSC for some patients, thereby likely obtaining more accurate SUV_mean_, MATV and TLG for some lesions. However, the use of these parameters was considered not feasible for reuse of baseline VOI due to image artefacts that were observed for some patients in the registered images. Only those parameters were used that showed a high DSC without any image artefacts, but this limits the flexibility of Elastix that may be required for some types of lesions. Classification of AUC was more affected by small lesions than classifications of other quantitative measures. An explanation for this is that AUC is ultimately dependent upon intensity histograms derived from individual tumours [23]. Therefore, tumour volumes should be sufficiently large to obtain valid results for AUC [24], [25].

Another high uptake area or lesion close to the target lesion can cause potential outliers for A50, as illustrated in figure 2. This bone lesion showed a decrease in SUV_max_ from 8.0 to 3.0 g/ml. The resulting SUV_max_ was close to the [^18^F]FDG uptake of the surrounding bone tissue, causing A50 to delineate a larger fraction of the bone. Nevertheless, this large increase in MATV (447%) was only 2.8 ml, thereby not classified as PMD. However, for the lesion depicted in figure 7, this did result in the inclusion of a nearby lesion and was therefore erroneously classified as a PMD.

Tumours with heterogeneous tracer uptake affect threshold-based delineation methods such as A50 [26]. For the image registration strategies, all PET-based image registration strategies used in this study measure similarity by maximizing normalized cross correlation [27]. Other similarity measures, such as normalized mutual information, might more appropriate for tumours that show (increased) tracer uptake heterogeneity. However, DSC for the two lesions that showed (increased) tracer uptake heterogeneity were lower for mutual information (0.31 and 0.26) compared to normalized cross correlation (0.37 and 0.35, data not shown), indicating that mutual information might not be more appropriate for tumours that show (increased) tracer uptake heterogeneity than normalized cross correlation.

For SUV_mean_, MATV, TLG and/or AUC, some lesions (three that affected two or more quantitative measures, and seven that affected AUC alone) showed deviating response classification when obtained with non-rigid PET and/or CTPET registration compared to A50 for late response assessments. These lesions showed a larger or smaller VOI for A50 compared to those obtained using non-rigid PET or CTPET registration. The difference in VOI between those obtained using A50 and non-rigid PET or CTPET registration could not be explained by the presence of high uptake area or lesion close to the target lesion. Possible scenarios include either the VOI obtained using A50 were larger or smaller because of the decrease/increase in SUV_max_, or the VOI obtained using non-rigid PET or CTPET registration were smaller or larger because of used similarity measure or the limited parameters used for the registration software (Elastix). Which VOI is more predictive can only be determined by future studies that correlate quantitative measures derived from each method to patient survival data. Therefore, for quantitative parameters such as SUV_mean_, TLG, MATV and AUC, future studies should be performed to further validate the use of non-rigid PET or CTPET registration for response classifications and correlating these to survival data. The fact that more deviating classifications were observed for AUC than for other quantitative measures may be explained by the higher sensitivity of AUC for differences in VOI placement/delineation compared to other metrics (i.e. SUV_max_, SUV_peak_ or even SUV_mean_, TLG and MATV). This indicates that any results on heterogeneity measures should be carefully checked for errors in tumor delineation or VOI placements. Recently, it has been shown that AUC is less sensitive to the type of tumor delineation compared to other (more local or regional) tracer uptake heterogeneity measures [28]. This may suggest that the performance for CTPET or PET registration may not be adequate enough for quantification of changes in global tracer uptake heterogeneity.

### Limitations

One limitation of this study is that the interval between [^18^F]FDG administration and the start of acquisition between subjects was 84±32 min, i.e. a fairly large inter-subject variability. The European Association of Nuclear Medicine (EANM) guidelines for quantitative [^18^F]FDG PET/CT studies [12] emphasize that the recommended scan time should be 60 min post injection and the same interval (tolerance ±5 min) should be applied in the context of therapy response assessments. Note, however, that this study occurred prior to the EANM guidelines and the sites were asked to scan at 60±10 min and then at the same time ±15 min for next scan. For most patients, the difference in scan time between baseline and response scan was small (i.e. 8±6 min). Only two patients showed a large difference in this interval (i.e. 88±18 min). As previously shown by Cheng et al. [29] the expected [^18^F]FDG uptake in the background surrounding a lesion may vary significantly at different imaging time points. Therefore, the variability in scan time between baseline and response scan is expected to have affected the observed absolute SUV and response values based on relative SUV changes, at least for these two patients. This would have been a serious limitation when the results would have been correlated with patient survival data and both patients should then have been excluded from the study. However, in this study, both A50 and the various registration strategies use the same input data and only differences between these methods are investigated. Furthermore, both methods are less sensitive for changes in contrast. All PET-based registration strategies use normalized cross correlation as a similarity metric that compensates for a (global) change in contrast. In addition, A50 is able to adapt its threshold relative to the local average background and is therefore less sensitive for a change in local contrast [3], [30]. Out of all five lesions identified within the two patients that showed a large deviation in scan time between baseline and response scan, only one lesion (figure 8) showed differences in response classification between A50 and the registration strategies, but this difference was likely caused by heterogeneous tracer uptake within the lesion. It is therefore expected that the difference in scan time between baseline and response scans had no effect on results presented in this study.

Another limitation of this study is the lack of correlative data, e.g. patient group survival data. As discussed earlier, both A50 as well as the proposed registration strategies have limitations and therefore this comparison can only provide limited conclusions. However, both methods use A50 as a common method to delineate VOI and therefore the comparison lies merely in the effect of reusing the baseline VOI after registration as opposed to independently delineating the VOI in all response scans. In addition, although there is no consensus on which (semi-)automatic delineation method to use in response monitoring studies, A50 has been shown to be an accurate and reproducible method to define VOI [3]–[6], [30].

## Conclusions

Non-rigid PET and CTPET image registration may be used to classify response based on SUV_max_ and SUV_peak_. For MATV, SUV_mean_, TLG and AUC future studies should be able to assess which method is valid for response evaluations by correlation with survival data.
